# Supplementary material for: Skewed logit model for analyzing correlated infant morbidity data
Source: PLoS One. 2021 Feb 8;16(2):e0246269. doi: 10.1371/journal.pone.0246269 (PMC7870004; doi:10.1371/journal.pone.0246269)
Supplement: Attachment — Submitted filename: plos_one_jul_20.pdf [file pone.0246269.s001.pdf]

The authors analyze the effect of bacterial vaginosis on infant morbidity using longitudinal subject measurements. They also measure the interaction of this effect with time. Such an analysis is often carried out using GEEs. Since the incremental effect of vaginosis on the probability of infant morbidity may not be largest for probabilities near 1/2, the authors suggest using a scobit link rather than the more common logit or probit links. The paper is written clearly, makes a reasonable methodological suggestion, and is of practical consequence. Some relatively minor issues are mentioned below.

1. Line 51 (also in abstract) "A critical issue of interest could be how we can model a binomial outcome that longitudinally violates symmetry." I think the authors need to be clearer early on what they mean by "symmetry." It wasn't clear until much later that they meant that the sensitivity to changes in the independent variable isn't maximized at 1/2.

2. The application of the scobit method to the data should include some justification based on the data, before seeing results. I.e., why do the authors find that a skewed S-curve is more appropriate to the data than a standard S-curve. Right now, the authors suggest they use the scobit link because it leads to a significant result whereas the logit does not, which is unreliable inference. In the authors' defense, the skewed family of S-curve they consider contains the standard S-curve. The larger family is significant whereas the subfamily (non-skewed logit) is non significant, some evidence that the latter is a bad fit.

3. It would be nice to see simulations, examples showing that the scobit links gives valid inference where the probit does not. On the other hand, the proposal is modest, so simulations are not as necessary.

4. Line 139 "Application to the Nairobi study infant morbidity dataset demonstrated that the SGEE outperforms the standard GEE in detecting a significant interaction between time and BV." The reasoning seems circular. Has it already been established that there is a significant interaction? This fact is being used as a "gold standard" by which to judge the proposed method, so it should be established by means other than the proposed method. Otherwise, simply assert that the proposed method finds a significant association, the standard method does not.

5. There should be a few sentences about the randomization. Data from between 1 and 6 visits are recorded. Could the variation in the visits be associated with the outcome? That would affect causal conclusions such as "Accelerated programs promoting access to BV treatment . . . may prove useful in reducing the incidence of infant morbidity in Kenya."

6. Line 115 "The reasons for considering these is that using a robust sandwich estimator implies that even if the correlation structure is misspecified, the parameter estimates remain valid." That the estimator is robust doesn't seem like a reason to consider several different then covariance models.

The usual argument is just that if correct model is chosen, the estimator is efficient.

7. Line 152, left out time subscript t in definition of Y_i

8. Line 161, Φ is the gaussian CDF? Then this looks like a probit not logit. Or is the claim that the probit on this transformed data is the same as a logit? It is difficult to interpret this passage with the undefined notation σ_v etc.

9. Line 218, "scobit" used without definition. In any event, it should probably be mentioned much earlier, to tie the method with familiar work.

10. Line 163, the cited authors differ from bibliography entry

11. Line 429 "controlled for other covariates" Should this say "controlling for", or maybe dialect variation.
