# Supplementary material for: Skewed logit model for analyzing correlated infant morbidity data
Source: PLoS One. 2021 Feb 8;16(2):e0246269. doi: 10.1371/journal.pone.0246269 (PMC7870004; doi:10.1371/journal.pone.0246269)
Supplement: Attachment — Submitted filename: Mwenda_feedback_27102020.pdf [file pone.0246269.s002.pdf]

Feed back to reviewers comments

To
Musie Ghebremichael
Academic Editor
PLOS ONE

Skewed-Logit model for Analyzing Correlated Infants morbidity data

Responses to the reviewers and additional comments on paper originally submitted as "Time Effects of Bacterial Vaginosis on Infant Morbidities in Kenya Assessed using Modified Skewed Logit Generalized Estimating Equations", and changed to "Skewed-Logit model for Analyzing Correlated Infants morbidity data"

There was a suggestion to change this topic to the above for simplicity and clarity

I have used the plos latex template to standardize the equations and lattef diff to track changes

General Comments

1. Please ensure that your manuscript meets PLOS ONE's style requirements, including those for file naming. The PLOS ONE style templates can be found at https://journals.PLOSOnebody.pdf and https://journals.PLOSOneaffiliations.pdf

Response: I have adhered to the formating and added the authors and affiliations to the final manuscript

2. Please specify in your ethics statement whether participant consent was written or verbal. If verbal, please also specify: 1) whether the ethics committee approved the verbal consent procedure, 2) why written consent could not be obtained, and 3) how verbal consent was recorded.

Response: The response was verbal and both the university of Nairobi and university of Washington approved

3. We note that you have indicated that data from this study are available upon request. PLOS only allows data to be available upon request if there are legal or ethical restrictions on sharing data publicly.

Response: We have anonymized the dataset and have provided it for replication purpose. Data are shared in Fig share and can be accessed using the following link. (`https://osf.io/yfzw5`) for data and r code at OSF public site

Reviewer 1 Comments

The main focus of this article is to implement a non-standard GLM/GEE method to analyze the infant morbidity and mortality, due to skewness of data. The authors incorporate Burr Type X (generalized Rayleigh) distribution and geeM package in R to achieve this goal.

1.My main concern is with the Methodology. I recommend that the authors focus on how the statistical analysis was implemented in geeM package of R, rather than going over the general technical details (see below). For this, the authors should understand the geeM package better, in order to make clearer and better technical arguments. In particular, the article will be more attractive if the authors include the R codes, either in the text or in the appendix/supplemental material.

Response: Thanks for pointing this out. We have read and referenced the works of (McDaniel et al., 2013). on (page 181) of their published paper gives drawback of the standard methods as " it does not allow the user to create and specify his own link and variance functions, and it is not easy for a programmer with only modest programming skills to alter the code for methodological developments related to or extending GEE." our main focus is on the flexibility of creating our own link functions, as an extension of methods proposed by (Nagler, 1994), and implemented in GLM. This work was demonstrating the use of that skewness parameter by implementing it into GEE.

2.For specific comments, there are instances where things could be eliminated or moved to the appendix. For example, the explanations from line 244 to 280 are standard in GLM and can be found in many books and articles, so the authors can just give a reference without having to explain in 3 pages.

Response: Thankyou for highlighting this. We have removed the details on β's estimation and refered readers to works of (Hardin, 2013) book chapter 1-3

Also, the notations are very inconsistent throughout. The both the X's and Y's are sometimes capital letters and sometimes lower case letters and sometimes bold letters; in both cases the subscripts are either inconsistent or non-existent. In addition, authors sometimes use X^T and other times X' to denote the X transpose. More seriously, the formulas (3) and (4), as well as the formula on line 309, are incorrect. These inconsistencies make the paper very difficult to read and make sense.

Response: Thankyou for noting this. We have carefully reworked our equations using latex, which gives us more control on how we write. All our equations are now updated and reviewed indepedently by a different statistician

Again, the authors must take care to make the technical arguments coherent and sound. By focusing more on understanding and implementing the R package that actually perform the analysis, the hope is that the paper will become more consistent and practical.

Response: Thank you for this comment, we have updated our arguments in the text.We have also have had our work edited by Editage, who are professional in enhancing manuscript flow and that the paper is free from any grammatical errors

Reviewer 2 Comments

The authors analyze the effect of bacterial vaginosis on infant morbidity using longitudinal subject measurements. They also measure the interaction of this effect with time. Such an analysis is often carried out using GEEs. Since the incremental effect of vaginosis on the probability of infant morbidity may not be largest for probabilities near 1/2, the authors suggest using a scobit link rather than the more common logit or probit links. The paper is written clearly,makes a reasonable methodological suggestion, and is of practical consequence.Some relatively minor issues are mentioned below.

Line 51 (also in abstract) A critical issue of interest could be how we can model a binomial outcome that longitudinally violates symmetry. I think the authors need to be clearer early on what they mean by symmetry.It wasnt clear until much later that they meant that the sensitivity to changes in the independent variable isnt maximized at 1/2.

Response: we have added the sentence "For example, for the Bernoulli distribution, binary asymmetry is defined as the sensitivity to changes in the independent variable that is not maximized at 0.5" in the introduction to bring out clarity of assymetry

The application of the scobit method to the data should include some justification based on the data, before seeing results. I.e., why do the authors find that a skewed S-curve is more appropriate to the data than a standard S-curve. Right now, the authors suggest they use the scobit link because it leads to a significant result whereas the logit does not,which is unreliable inference. In the authors defense, the skewed family of S-curve they consider contains the standard S-curve. The larger family is significant whereas the subfamily (non-skewed logit) is non significant,some evidence that the latter is a bad fit.

Response: Thankyou for this feedback. Our motivation for using the skewed logit, is based by the assumptions of asymmetry in the binary response demonstrated by (Nagler, 1994) and (Tay, 2016). Our data as attached clearly meets the requirements for binary violation since the final yes/no for morbidity is constructed from a continuous final score derived from yes/no for different morbidities. for example, a woman would go for a scheduled visit and asked whether the child had any sickness within that month. A tally of all illnesses recorded during that month was recorded(hereby refered to as score). The symmetry assumption was violated in the extremely sick(those with number of morbidities exceeding 1 at any given month) and the Skewed Logit fitted our data well. This justifies the use of Skewed logit based on our data before the analysis. Morbidities severity models were estimated using illness data from HIV positive women who were tested for BV nairobi

It would be nice to see simulations, examples showing that the scobit links gives valid inference where the probit does not. On the other hand, the proposal is modest, so simulations are not as necessary.

Response: Thanks for this comment, some simulations have already been provided by (Nagler, 1994) on his paper "Scobit: An Alternative Estimator to Logit and Probit" on 238-240, that proved SL was superior in some instances

Line 139 Application to the Nairobi study infant morbidity dataset demonstrated that the SGEE outperforms the standard GEE in detecting a significant interaction between time and BV. The reasoning seems circular.Has it already been established that there is a significant interaction This fact is being used as a gold standard by which to judge the proposed method, so it should be established by means other than the proposed method. Otherwise, simply assert that the proposed method finds a significant association, the standard method does not.

Response: Thanks for pointing this. We have added a sentence "When we chose a p-value =0.05 level of significance, parameter estimates from the standard GEE were not significant. In this case, only time and gender were significant. However, when using the skewed logit GEE, gender, time, BV, and the interaction between time and BV were significant." in our result section to capture this comment.

There should be a few sentences about the randomization. Data from between 1 and 6 visits are recorded. Could the variation in the visits be associated with the outcome That would affect causal conclusions such as Accelerated programs promoting access to BV treatment . . . mayprove useful in reducing the incidence of infant morbidity in Kenya.

Response: Thankyou for this. We have explained in details about randomization in a new section named "materials and methods, under data"

Line 115 The reasons for considering these is that using a robust sandwich estimator implies that even if the correlation structure is misspecified, the parameter estimates remain valid. That the estimator is robust doesn't seem like a reason to consider several different then covariance models.The usual argument is just that if correct model is chosen, the estimatoris efficient.

Response: Thankyou for the comment. I have re edited that part and added a new line " Liang and Zeger stated that the mis-specification of $\mathbf{R}_i(\boldsymbol{\rho})$ only affects the efficiency of the $\hat{\boldsymbol{\beta}}$ and $\hat{\boldsymbol{\beta}}$ is robust against mis-specification"

Line 152, left out $time_t$ subscripttin definition ofY_i

Response: Thanks. We have reworked all equations to conform to the PLOS standards.

Line 161, Φ is the gaussian CDF Then this looks like a probit not logit.Or is the claim that the probit on this transformed data is the same as alogit It is difficult to interpret this passage with the undefined notation $\sigma_v$vetc.

Response: Thanks. We have reworked all equations to conform

Line 218,'scobit' used without definition. In any event, it should probablybe mentioned much earlier, to tie the method with familiar work.

Response:thankyou for pointing out this. We have replaced 'scobit' with a common name, skewed logit which is used in the whole manuscript and SGEE with SL-GEE used in the table

Line 163, the cited authors differ from bibliography entry

Response: Thanks for that. We have removed that part as suggested by reviewer 2 and ensured all authors cited appear in the bibliography.

Line 429 controlled for other covariates Should this say 'controlling for',or maybe dialect variation.

Response: We have corrected the sentence to read "Not all covariates included in our study were statistically significant at $p = 0.05$. Nonetheless, their coefficient sign could assist in detecting a trend of association with the response."

Reviewer 3 Comments

This study uses data from the Nairobi infant morbidity study to evaluate the effects of time and bacterial vaginosis (BV) on morbidity among infants born to women infected with HIV-1. The authors compared the results of their data analysis using a traditional generalized estimating equations (GEE) model versus their newly-developed skewed generalized estimating equations (SGEE) model, which extends the GEE framework to accommodate possible asymmetry.

It is unclear if the authors are presenting a novel statistical method versus a novel application of an existing method to shed light on the effects of time and VB on infant mortality. Note that lines 182-184 suggest the former while lines 137-139 and 455-456 suggest the latter.

Response: Thankyou for pointing this. We have edited the lines 137-139 to read "motivated by the fact that this methodology has few applications in longitudinal corelated data".

If the formulation of their SGEE model is novel, it would be helpful to formally validate the utility of this method (versus GEE) is the presence of asymmetry using simulation studies. If this work has already been done and published elsewhere, and the authors are presenting a novel application of this method, Im wondering if it would be possible to exclude the majority of the technical information contained in the methods section (or at least move it to an appendix), and report a validated goodness of fit/model comparison measure for the GEE versus SGEE models applied to their data.

Response: Thankyou for this. We have deleted most of the technical details and provided link to the published work. we have maintained the minimal number of statistical equations

Line 40: Im not sure what is meant by distorted in this sentence. Do the authors mean transformed

Response: Thankyou for this. We have changed the sentence to replace distorted with "transformed"

Lines 42-44: A typical assumption for the distribution of the error term in logistic analysis is the logistic function that is commonly applied to data with standard binomial distributions. I believe the phrasing that suggests logistic regression models have error variables that follow a logistic distribution is characteristic of discrete choice models, primarily used by economists. Given that this paper involves a biomedical application (and will likely appeal to a clinical audience) it would be helpful to rephrase.

Response: Thanks for this point. we have re edited to read "For example, imbalances can occur in binary response data, when symmetry is violated. There are two types of models which are typically employed to analyze data in these scenarios - 1) logit and 2) probit models. Logit models have error variables that follow a logistic distribution and this type of model is considered to be characteristic of discrete choice models. The probit model uses the cumulative standard normal distribution function and assumes the error term is normally distributed. Although this assumption is viewed as a reasonable compromise to achieve mathematical simplicity and parsimonious results, its suitability has been doubted of late."

Line 58: The supporting literature has shown that this could be inefficient for parameter estimation in some settings could use a supporting reference.

Response: Thanks for this. We have provided three references by citing works by (Nagler, 1994; Prentice, 1976; Tay, 2016)

The notation introduced at the beginning of the methods section is ambiguous. For example, line 146 introduces nj as the number of observations observed for subject i. Later, the notation ni is used to denote the dimension of the covariate matrix and response vector. Repeated use of the same letter with varying subscripts is confusing. Elsewhere (line 150) the authors use Yij to (presumably) denote the jth observation for subject i, but then two lines later (line 152) define Yi as an indicator for subject is morbidity without reference to a particular time. This suggests that the authors are modeling a univariate response. Was the second subscript j left off in line 152

Response: We have edited all our equations in LATEX to have flexibility and consistency.

Line 149: I'm not sure what a matrix-vector is Do they mean covariate matrix

Response: We have changed to covariate matrix

Line 188: Y_0 is not defined.

Response: Thanks.We have edited as part of equations

Line 342: It would be good to explicitly state how the response variable was categorized (e.g. 0 vs at least 1 morbidity at a given monthly visit)

Response: We have explained in details on a new section **Materials and methods, data** "From the scores, we created a binary response; Those who had illness(mild and severe) and those who didn't have any illness within each given month from month 1 to 6, in order to have a population average interpretation"

Line 352: Since the authors are interested in comparing morbidity by month among infants born to mothers with vs without BV, it would be easier to digest Table 1 if it presented the percentage of infants with at least one morbidity conditional on BV status during each month. For example, during month 1 a direct calculation of the 115/(115+33)=78% of infants experiencing a morbidity within the BV-present group vs the 85/(85+94)=47% of infants experiencing a morbidity within the BV-absent group highlights the relevant comparison more clearly.

Response:. Thankyou for this point. We have created a new table "BV with morbidity incidences reported from month one to six for both BV-exposed and unexposed babies in the Nairobi data survey" to serve 2 purposes. 1) show the assymetry in the responses 2)present the percentages of infant with atleast one morbidity as you have suggested.

Line 357: important to examine the effect of BV on child morbidity should add over time

Response: We have added the statement to read "We sought to assess whether children born to women who tested positive for BV were more likely to have a higher morbidity incidence than their counterparts and if the effects would change with time"

Line 358: Was data from birth through 6 months used in this analysis (as stated in line 325) Here t=1,2,,6 suggests data from birth was not used.

Response: We have edited our table and cited our previous work and other literature, which found no significance between morbidity incidences and BV at birth. we have added this sentence "Data on morbidities from birth were included in the month 1 tally, and not as an independent time period, since morbidities due to BV on neonates was found to be insignificant in previous research".

Line 364: We wanted to show that our model fits better for binary data that violate symmetry. As noted above, this general goal is better achieved using simulations.

Response: We have cited literature by (Nagler, 1994) that have supported this through simulations, and as adviced by reviewer 2, we have cited the literature in support. we have replaced the statements to read "In this paper, we were interested in comparing two models; the SL-GEE and the standard GEE when the response is assumed to be asymmetric"

Line 365: We tested different correlation structures for comparison purposes; however, because our model was concerned with the time effect, we used the AR(1) output for interpretation This point could be clarified and could use some elaboration. How did the correlation structures compare overall How is the use of AR(1) beneficial regarding modeling the effect of time in this application Is it because measurements taken further apart have lower correlation according to the pattern imposed by AR(1)

Response: Thanks for this. We have edited our sentence to read "We assessed different correlation structures and all our parameters estimate were within the acceptable standard errors. However, measurements taken apart from the same individual have lower correlation, follow a pattern imposed by AR(1), thus for interpretation of our work it was adopted."

Line 381: Where is -0.372 coming from The coefficient for sex using the SGEE model with AR(1) is -0.369. Also, it would be helpful to explicitly note that this is an odds ratio (line 380).

Response: Thanks for noting this. We have re run our model again and the correct value is -0.69. we have also ensured our interpretations are based on Odds ratio as suggested.

Line 381: Those with BV had a 4.48 odds of morbidity compared with their counterparts is this the OR at birth I am not sure how this OR is interpreted because time is coded as t = 1, 2, ..,6 (358) and there is an interaction term. Did the authors use data at birth (time =0)

Response: We have edited our statements and reformated our table and since there was no significant association of BV with neonates morbidities, we removed this part from the table. we didn't analyse morbidities at birth independently. However, the morbidities were recorded and tallied in the first month

Line 386: The authors should explicitly state that the quantity they are using in an OR.

Response: Thanks for this. We have replaced the odds with OR

Line 392 (and Table 3): Again, was data from birth used in this application or is the effect being extrapolated (again, line 358 suggests outcome measurements were at months 1-6)

Response: We have edited to reflect from month 1 to month 6

Line 395: Where is the quantity 2.72 coming from Isnt the OR 3.37 And should 1.04 be 1.11

Response: We have re edited to capture 3.37 and 1.11. it was an error of commision. our sentence now reads "We have higher morbidity effects from month 1 and the effects decrease with time. For example, if we compare month 1 and month 5 we can conclude that at month 1, the OR of having morbidity incidences are 3.37 times for children whose mothers had BV compared to those who didn't have. At month 5 the OR decrease to 1.11 for mothers who tested positive for BV versus those that tested negative"

Line 410: Best to avoid causal language such as proved.

Response: Thanks for pointing this. We have replaced proved with found out. our reconstructed sentence now reads "...commonly neglected 'minor diseases' and found out they should not be ignored at the expense..."

Line 419: Again, avoid using the word proven.

Response: Thanks for pointing this. We have replaced proven with found out

Line 451: In addition, our study found a decline in the number of morbidities from birth Table 1 suggests that there was a decline in morbidity (defined as no morbidity vs at least 1 morbidity) over time among infants in the BV-positive group but an increase in morbidity over time among infants in the BV-negative group.

Response: Thanks for pointing this. We have re-edited our work to read "Past work by Verma *et al.* indicated an increase in the number of illnesses during infant growth when **?**. This is in contrast with what was reported in table **??**, which shows only an insignificant decline among all the infants(5%), from a high of 61% to a low of 56%. To be more precise, considering the BV-exposed group, there was a huge decline of 25%, from a high of 78% in the first month to 53% in the sixth month. However, in the non-exposed group, there was a slow increase, whereby the number of morbidities observed increased from 47% in month one to 58% in month six. However, this study was based on the general population of the infants, without factoring in any other factors defining the exposed group or applying any randomization. "

Lines 461-463: Asymmetry in the binary outcome is a phenomenon that should be appropriately accounted for in analytical models to avoid biases in final parameter estimates, as has been established in Table 2 Its a stretch to draw this conclusion based on an inspection of p-values from just one application. The authors should cite simulation studies using their exact formulation of the SGEE model or validated measures of fit applied to this data.

Response: Thanks for pointing this. We have added more citations to support our paper and re written the sentence as " Asymmetry in the binary outcome is a phenomenon that should be appropriately accounted for in analytical models to avoid biases in final parameter estimates, as has been established in this paper and

other works (Coelho et al., 2013; Nagler, 1994; Prentice, 1976; Tay, 2016). We have also added a table on the variances ratio computed from among the correlation structures

Line 464: I believe the term converges should be replaced with is equivalent

Response: We have replaced to read "skewed logit density is equivalent to a logit density"

References

Coelho, R., Infante, P., and Santos, M. N. (2013). Application of generalized linear models and generalized estimation equations to model at-haulback mortality of blue sharks captured in a pelagic longline fishery in the atlantic ocean. *Fisheries Research*, 145:66 – 75.

Hardin, J. W. (2013). *Generalized estimating equations. Hardin, Hardin,.* CRC Press, Boca Raton, Fla.

McDaniel, L. S., Henderson, N. C., and Rathouz, P. J. (2013). Fast pure r implementation of gee: Application of the matrix package. *The R journal*, 5:181–187.

Mwenda, N., Nduati, R., Kosgey, M., and Kerich, G. (2020). Morbidities and mortality among infants of hiv-1-infected mothers with bacterial vaginosis in kenya.

Nagler, J. (1994). Scobit: An alternative estimator to logit and probit. *American Journal of Political Science*, 38(1):230–255.

Nduati, R., John, G., Mbori-Ngacha, D., Richardson, B., Overbaugh, J., Mwatha, A., Ndinya-Achola, J., Bwayo, J., Onyango, F. E., Hughes, J., and Kreiss, J. (2000). Effect of breastfeeding and formula feeding on transmission of hiv-1: a randomized clinical trial. *JAMA*, 283:1167–74.

Prentice, R. L. (1976). A generalization of the probit and logit methods for dose response curves. *Biometrics*, 32:761–8.

Tay, R. (2016). Comparison of the binary logistic and skewed logistic (scobit) models of injury severity in motor vehicle collisions. *Accident Analysis and Prevention*, 88:52 – 55.
