# Supplementary material for: Skewed logit model for analyzing correlated infant morbidity data
Source: PLoS One. 2021 Feb 8;16(2):e0246269. doi: 10.1371/journal.pone.0246269 (PMC7870004; doi:10.1371/journal.pone.0246269)
Supplement: Attachment — Submitted filename: response to reviewers.pdf [file pone.0246269.s003.pdf]

Feed back to reviewers comments

To
Musie Ghebremichael
Academic Editor
PLOS ONE

Skewed-Logit model for Analyzing Correlated Infants morbidity data

General Comments

2. Is the manuscript technically sound, and do the data support the conclusions?

Response: We thank the reviewers for this comment and finding merit in our work.The previous work had suggested major revisions and we have worked hard to respond to every question.

3. Has the statistical analysis been performed appropriately and rigorously?

Response: We acknowledge the kind response on our statistical analysis, indeed we had our methods checked by an experienced statistician.

4. Have the authors made all data underlying the findings in their manuscript fully available?

Response: We have anonymized the dataset and have provided it for replication purpose.As advised by PLOS one, data have been shared and achieved in Fig share and can be accessed using the following link. (`https://osf.io/yfzw5`) for data and r code at OSF public site

5. Is the manuscript presented in an intelligible fashion and written in standard English?

Response: Thanks for pointing this out. We have had our work Edited by Editage professionals and provided the editing certificate.

Reviewer 1 Comments

For the GEE model, it would be helpful to show additional details of model selection. Authors may consider using QIC or QICu functions in R package MuMIn

Response: Thankyou for highlighting this. We have reviewed the methodology underlying QIC and QICu framework as provided by Hardin (2013), in their book Generelized Estimating Equations. On page 163-171, and they explain that the framework is good in model selection only when (1) a researcher is seeking to find the best correlation structure among the competing ones and (2) when models have different number of covariates and a researcher wants to choose the most parsimonous.

However, our approach is comparing the Logit and the Skewed Logit, where the later factors in binary skeweness. In this scenario, both the QIC and the QICu are not appropriate for model selection. this is because, in the book of Hardin (2013) on page 171, they point out that "This criterion **(QIC and QICu)** is meant as a guide for choosing between models when no scientific knowledge would guide the researcher to a preference." For our modeling approach, a large body of literature supports maternal Bacterial Vaginosis and time effect on infant morbidity, therefore we prefer output from the skewed Logit since it was able to bring out this important association.

On page 18, equation (9), there should only be 6 main effects. I think β_4 is redundant. Also, for the interaction term, it is more customary to write β_{15} with (BV $\times$) time

Response: Thank you for noting this. We have removed the redundant $\beta_4 BV_i$ to ensure we only have 6 betas.

Again, providing the computer code will be very much appreciated.

Response: We have anonymized the dataset and have provided it for replication purpose.As advised by PLOS one, data have been shared and achieved in Fig share and can be accessed using the following link. (`https://osf.io/yfzw5`) for data and r code at OSF public site

Reviewer 2 Comments

"The application of the scobit method to the data should include some justification based on the data, before seeing results ..."

Response: Thank you for this. We have captured this in the Data section, the last paragraph that reads " A standard questionnaire developed by the principal investigator of the study to identify illnesses was completed for both the mother and the child. This was achieved using a 19-item yes/no morbidity questionnaire which purports to measure health status of an infant. The total score of the questionnaire is computed as the count of all the "yes" responses. There were a total of 1962 observations from 327 pairs of mothers and babies. From the total score, we created a binary response of; (1) those who did not have any illness and (2) those who had either minimal or severe illnesses. **Table 1** presents an initial exploratory analysis used to identify the asymmetry in the total responses for each month. **This evidence of asymmetry justifies the use of the skewed logit model**."

References

Hardin, J. W. (2013). *Generalized estimating equations. Hardin, Hardin,.* CRC Press, Boca Raton, Fla.
